# Adaptation pathways of global wheat production: Importance of strategic adaptation to climate change

**DOI:** 10.1038/srep14312

**Published:** 2015-09-16

**Authors:** Akemi Tanaka, Kiyoshi Takahashi, Yuji Masutomi, Naota Hanasaki, Yasuaki Hijioka, Hideo Shiogama, Yasuhiro Yamanaka

**Affiliations:** 1 Center for Social and Environmental Systems Research, National Institute for Environmental Studies, 16-2 Onogawa, Tsukuba, Ibaraki 305-8506, Japan; 2 College of Agriculture, Ibaraki University, 3-21-1 Chuo, Ami, Inashiki, Ibaraki 300-0393, Japan; 3 Center for Global Environmental Research, National Institute for Environmental Studies, 16-2 Onogawa, Tsukuba, Ibaraki 305-8506, Japan; 4 Faculty of Environmental Earth Science, Hokkaido University, N10 W5, Kita-ku, Sapporo, Hokkaido 060-0810, Japan

## Abstract

Agricultural adaptation is necessary to reduce the negative impacts of climate change on crop yields and to maintain food production. However, few studies have assessed the course of adaptation along with the progress of climate change in each of the current major food producing countries. Adaptation pathways, which describe the temporal sequences of adaptations, are helpful for illustrating the timing and intensity of the adaptation required. Here we present adaptation pathways in the current major wheat-producing countries, based on sequential introduction of the minimum adaptation measures necessary to maintain current wheat yields through the 21st century. We considered two adaptation options: (i) expanding irrigation infrastructure; and (ii) switching crop varieties and developing new heat-tolerant varieties. We find that the adaptation pathways differ markedly among the countries. The adaptation pathways are sensitive to both the climate model uncertainty and natural variability of the climate system, and the degree of sensitivity differs among countries. Finally, the negative impacts of climate change could be moderated by implementing adaptations steadily according to forecasts of the necessary future adaptations, as compared to missing the appropriate timing to implement adaptations.

There are various options of agricultural adaptation to climate change. To reduce the negative impacts of climate change and obtain greater benefits, not only autonomous adaptations (e.g., shifting the planting date, switching crop varieties) but also planned adaptations that require substantial investment (e.g., development of new crop varieties, expanding irrigation infrastructure) are needed^1^,^2^. For example, the Canadian and U.S. governments have invested in research leading to many innovations in wheat varieties^3^. From the viewpoint of international food security, it is important to identify which regions in the world are vulnerable to climate change and in need of such investment for adaptation. Analyses of climate risks in multiple regions of the world were conducted in response to this concern^1^. In addition, it is also important to consider how major food-producing countries should adapt to climate change in order to maintain the food supply to meet global food demand.

Recent studies have analyzed climate-change impacts and the benefits of adaptation on crop yields^4^. However, many of them assessed the situation while assuming that the adaptations are fully implemented or not implemented at all, especially at the global scale, and that adaptation is completed immediately. Few studies have assessed the course of adaptation along with the progress of climate change in each of the current major food-producing countries. Information on the course of adaptation, which shows the timing and intensity of future adaptations, will be useful for adaptation planning. At least during the planning phase, adaptation steps should be regarded as irreversible so as not to spend funds in vain; and future adaptations must be forecast because substantial time is required from planning to the introduction and dissemination of an adaptation^5^. For example, a new crop variety usually takes between 8 and 20 yr to deliver^6^.

An effective way to examine the timing and intensity of adaptations is to describe the temporal sequences of the adaptations. The concept of describing a sequence of adaptation options over time, termed “adaptation pathways,” was recently proposed^7^,^8^ and applied to assessing regional adaptation^9^,^10^. In the previous studies, the adaptation pathways showed potential sequences of actions under climate change and social changes in order to explore robust adaptive plans. Here, however, we focus on describing adaptation pathways by sequential implementation of the minimum necessary adaptation. The resulting pathways reveal the timing and intensity of adaptations required to meet the challenges of climate change.

In this study, we present nation-wise adaptation pathways for the global production of wheat, which is a staple crop worldwide. We developed adaptation pathways for the current major wheat-producing countries (China, India, the United States, Russia, France, Canada, Germany, Turkey, and Pakistan)^11^ from the 2010 s (2011–2020) to 2090 s (2091–2100) based on sequential implementation of adaptation measures to maintain the levels of yield in the 1990 s (1991–2000). We employed multiple climate models to analyze the uncertainties of climate-change projections: MIROC-ESM^12^, MPI-ESM-LR^13^, and CSIRO-Mk3-6-0^14^ from the Coupled Model Intercomparison Project Phase 5 (CMIP5)^15^. We also used available model ensemble runs because the natural variability of the climate system is another source of uncertainty in addition to model uncertainty^16^,^17^; three runs each from MPI-ESM-LR and CSIRO-Mk3-6-0 were employed. We used all models and ensemble runs (i.e., 7 projections) for analyzing uncertainties arising from climate-change projections, and we primarily used MIROC-ESM in other analyses.

For MIROC-ESM, we used two extremely different radiative forcing scenarios of the Representative Concentration Pathways (RCPs): the RCP8.5 scenario^18^ and the RCP2.6 scenario^19^. The RCP8.5 scenario corresponds to the highest greenhouse gas emissions scenario, leading to the greatest increase in global mean temperature, whereas the RCP2.6 scenario corresponds to a mitigation scenario aiming to limit the increase in global mean temperature in the 21st century to 2 °C from the pre-industrial level. We used the RCP8.5 scenario throughout the analyses, and then used the RCP2.6 scenario for comparison. For the other models and ensemble runs, we used the RCP8.5 scenario.

We considered two adaptation options: (i) expanding irrigation infrastructure, with 13 intensity levels of adaptation (hereafter referred to as “adaptation levels”; see Methods); and (ii) switching crop varieties and developing new heat-tolerant varieties, with 6 adaptation levels. We estimated country-based yield for all combinations of adaptation option and adaptation level (hereafter referred to as “adaptation set”), and then developed adaptation pathways by implementing a stronger and minimum-necessary adaptation set to avoid a yield decrease from that of the 1990 s (hereafter “current yield”) in each decade (see Methods). When examining planned adaptation, we assumed that adaptation levels are irreversible: once a certain level of adaptation is taken, then the lower adaptation levels cannot be used. For simplicity, we did not consider adaptation costs or socio-economic changes such as the change in demand, economic growth, and technological progress. In order to construct adaptive plans optimized for each country, region-specific adaptive capacity and criteria of decision-making need to be considered, which is beyond the scope of this study.

## Results

### Adaptation pathways to maintain current wheat yields

**Figure 1:**
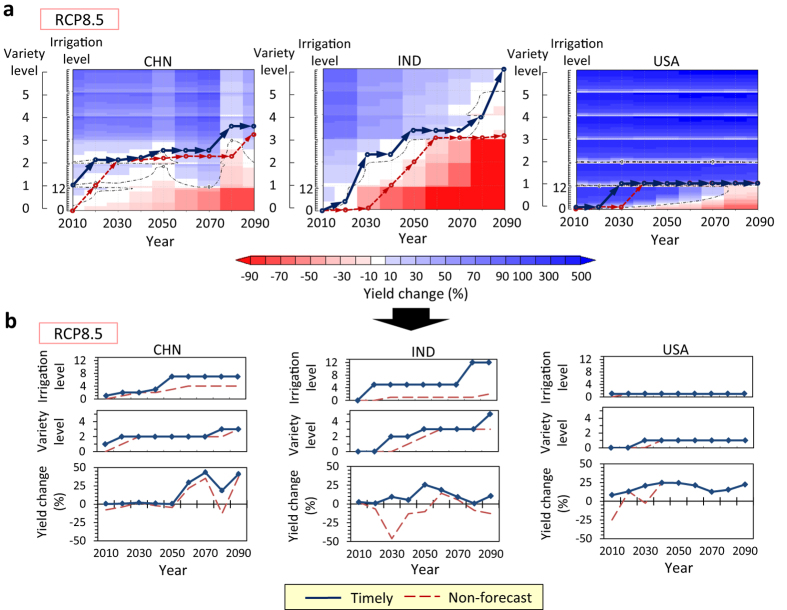
Adaptation pathways and yield change from the current level in the timely case and in the non-forecast case in China (CHN), India (IND), and the United States (USA) under the RCP8.5 scenario of the MIROC-ESM projection. (**a**), Time evolution of yield change for all adaptation sets (13 irrigation × 6 variety) and adaptation pathways. The thin black broken lines represent 0% change. (**b**), Adaptation pathways and yield change extracted from (**a**).

Without adaptation (i.e., Level 0 both for irrigation and crop variety), wheat yields will fall below the current level in most countries in the 21st century under the RCP8.5 scenario (Fig. 1a, Supplementary Fig. S2). This is common to all of the climate models and ensemble runs. We found that only a limited number of the adaptation sets could maintain wheat yield near the current level (yield change ≈ ± 10%); late implementation of adaptation leads to a large yield decrease, and early implementation leads to an unnecessary yield increase possibly linked with overinvestment (Fig. 1a).

**Figure 2:**
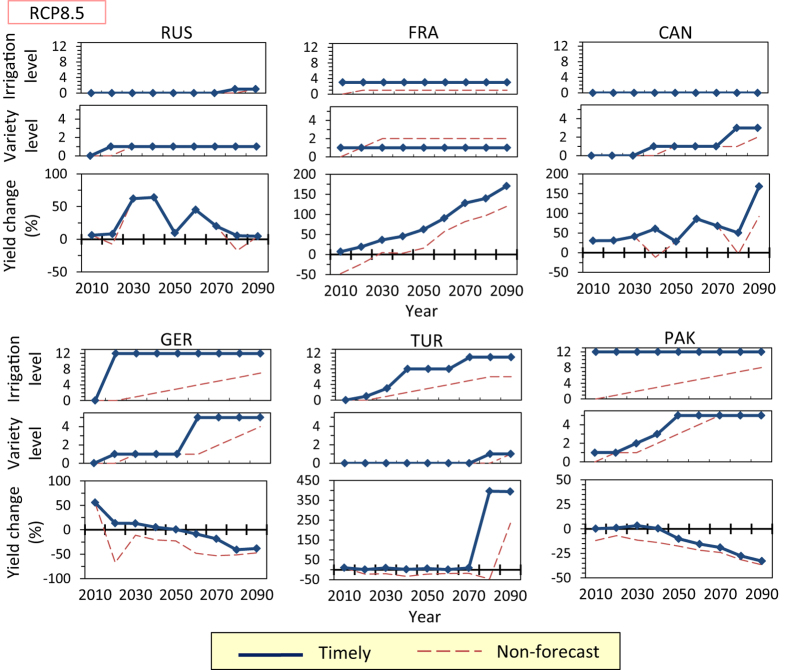
Adaptation pathways and yield change from the current level in the timely case and in the non-forecast case in Russia (RUS), France (FRA), Canada (CAN), Germany (GER), Turkey (TUR), and Pakistan (PAK) under the RCP8.5 scenario of the MIROC-ESM projection.

When the minimum-necessary adaptation set (i.e., the adaptation set that is expected to attain the minimum increase in yield from the current yield) is implemented incrementally in each decade, the adaptation pathways under the RCP8.5 scenario of the MIROC-ESM projection result in the dark blue lines in Figs 1 and 2. These pathways indicate the time sequences of necessary adaptation, and they are also assumed to be the results of timely implementation of necessary adaptation; thus, we refer to these pathways as the “timely case.” Adaptation pathways in the timely case are quite different among the countries with regard to the amount and timing of adaptation required (Figs 1,2). In China and India stronger adaptations will be required over time, whereas in the United States and France, the adaptation needs to be implemented only in the earlier decades and could be kept constant later. The pathways in the United States and Russia could maintain yields near the current level just by switching to existing crop varieties (crop variety Level 1) and increasing the irrigated area ratio by 10% (irrigation Level 1). In contrast, both new heat-tolerant varieties (i.e., crop variety Levels 2 to 5) and increasing the irrigated area ratio by ≥ 50% (i.e., irrigation Levels 5 to 12) will be required to maintain current yields in China, India, Germany, and Pakistan (see Methods). In China, India, and Pakistan, crop land in which the current irrigated area ratio is relatively high ( > ~50%) is widely distributed, such that the irrigated area ratio in the currently irrigated region may reach the upper limit (100%). Expansion of irrigation into current rain-fed crop lands (i.e., irrigation Levels 11 and 12) will be required in India and Pakistan (Figs 1,2). In Germany, most crop lands are rain-fed, but a substantial expansion of irrigation into the rain-fed areas will be required to maintain current yield (Fig. 2).

### Adaptation pathways developed based on multiple climate-change projections

**Figure 3:**
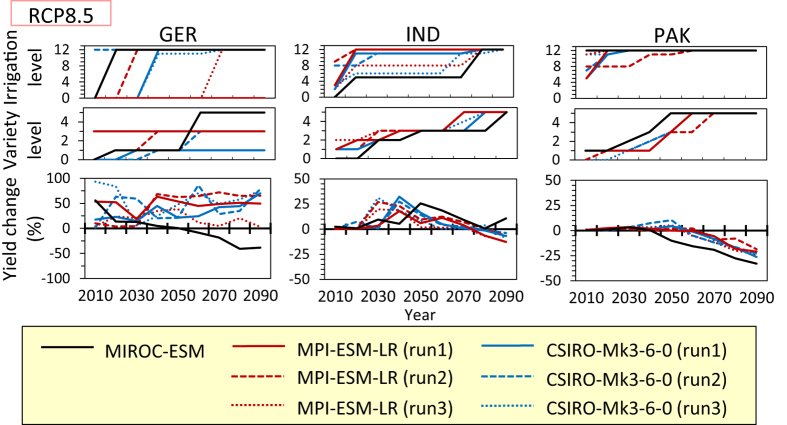
Adaptation pathways and yield change from the current level in the timely case of all the climate projections in Germany (GER), India (IND), and Pakistan (PAK) under the RCP8.5 scenario. The descriptions “run1”, “run2”, and “run3” correspond to r1i1p1, r2i1p1, and r3i1p1 of the CMIP5 simulations, respectively.

In addition to using the MIROC-ESM model, we also developed adaptation pathways in the timely case under the RCP8.5 scenario with the other climate models and ensemble runs. Although at first glance the pathways appear quite different among the models and ensemble runs, we did find some common features in the selected options in several countries (Fig. 3, Supplementary Fig. S3): (i) changing crop varieties is preferable to expanding irrigation in Russia and Canada, whereas a large expansion of irrigation is chosen in France and Turkey without the development of new crop varieties; (ii) high adaptation levels for both irrigation and crop variety are required in India; (iii) all models and ensemble runs projected that the wheat yield will fall below the current level in Pakistan even if the highest levels of adaptation are implemented in the late 21st century; and (iv) in Germany, though the wheat yield can be maintained via the adaptation through the 21st century, except under the MIROC-ESM projection, most projections indicate that irrigation needs to be expanded into current rain-fed crop land (i.e., irrigation Levels 11 and 12). (Note that because we assumed irrigation could be expanded into current rain-fed crop land only at irrigation Levels 11 and 12 [see Methods] and the maximum irrigated area ratio is limited [i.e., 50% at Level 12], necessary adaptation levels might be high in those regions where most crop lands are rain-fed, as in Germany).

The timing of the adaptation varies widely among the models and ensemble runs in these simulations. With regard to the benefit of adaptation, there are large uncertainties arising from climate model uncertainty and natural variability of the climate system (Supplementary Fig. S4), which leads to differences in the adaptation pathways among models and ensemble runs. For example, the attainable wheat yield for each adaptation level appears to be similar among the projections in India; but adaptation levels required to maintain the current wheat yield fluctuate due to natural variability, leading to differences in the adaptation pathways (Fig. 3). In Germany, both uncertainty caused by climate model and uncertainty caused by natural variability are quite large, leading to large differences both in adaptation pathways and in attainable wheat yields (Fig. 3).

### Effects of adaptation strategies and emissions scenarios

Because we considered multiple adaptation options (i.e., expansion of irrigation and crop variety alteration), multiple pathways of necessary adaptation could be described by prioritizing one of the adaptation options. In this section, we show effects of these adaptation prioritization strategies on the adaptation pathways, focusing on the results of MIROC-ESM. We also show the adaptation pathways under the different emissions scenario (i.e., the RCP2.6) based on the MIROC-ESM projection in the latter half of this section.

**Figure 4:**
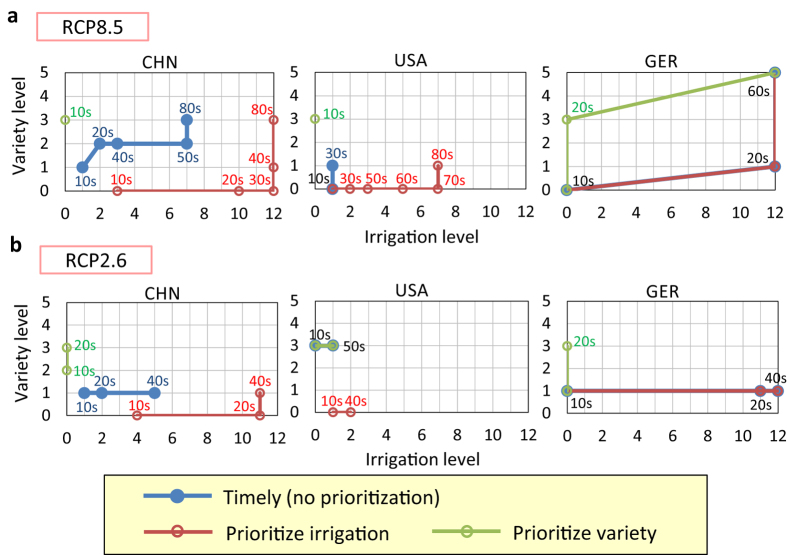
Adaptation levels of irrigation and crop variety for each decade from the 2010s to 2090 s (adaptation pathways) in three cases (timely, prioritize irrigation, and prioritize variety cases) under the MIROC-ESM projection. The countries shown are China (CHN), the United States (USA), and Germany (GER). The numerals within graphs indicate decades in the 21st century when the adaptation set is implemented (e.g., “10 s” represents the 2010 s). (**a**), Under the RCP8.5 scenario. (**b**), Under the RCP2.6 scenario.

The timely case previously mentioned is a non-prioritization case: the adaptation set that achieves the minimum increase in yield from the current level is chosen regardless of the kind of adaptation option. If either irrigation or crop varieties is preferentially chosen (the prioritized cases; see Methods), the resulting adaptation pathways differ from the timely case (Fig. 4a, Supplementary Fig. S5). For example, in the United States and Russia, although a combination of switching to existing crop varieties and a 10% increase in irrigation could maintain the current yields (timely case), introduction of new heat-tolerant crop varieties will be required if irrigation is not expanded (prioritize variety case). The current yield will be maintained by the adaptation assumed in this study in the timely case and/or prioritized cases in all countries except Germany and Pakistan under the RCP8.5 scenario (note that the wheat yield in Germany is projected to be maintained by the other climate models and ensemble runs; see the previous section). However, the adaptation pathways include a large expansion of irrigation or development of new-heat tolerant varieties in some cases, and the timing of the required adaptation varies with the prioritization of the adaptation options. Decision-makers have to examine adaptations by considering the country-specific adaptive capacity or strategy.

The necessary adaptation levels tend to be lower under the RCP2.6 scenario than under the RCP8.5 scenario, except in the United States and France (Fig. 4b, Supplementary Fig. S6). In Russia and Canada, the adaptation levels are lower and the timing of implementing adaptation is later than under the RCP8.5 scenario. In China, the yields could be maintained at the current level by switching to existing crop varieties and expanding the irrigated area ratio; the development of new varieties that is required under the RCP8.5 scenario may not be necessary under the RCP2.6 scenario. Unlike the RCP8.5 scenario, the yields could be maintained in Germany and Pakistan under the RCP2.6 scenario. If irrigation is expanded into current rain-fed crop lands (i.e., irrigation Levels 11 and 12) in Germany, the yield could be maintained just by switching to the existing crop varieties. In Pakistan, although new varieties (i.e., crop variety Levels 2 to 5) will be needed even if irrigation is expanded into currently rain-fed crop lands, the likelihood of maintaining the current yield by adaptation would be higher under the RCP2.6 scenario than under the RCP8.5 scenario. In these countries, future climate change will be particularly important for adaptation planning. In other words, climate mitigation policy matters more there. In the United States and France, the adaptation levels are not necessarily lower under the RCP2.6 scenario than under the RCP8.5 scenario. The possible reasons are that under the RCP2.6 scenario either a high level of irrigation or a high level of crop variety is selected in the early decades instead of a combination of adaptation options, and water stress would be higher under the RCP2.6 scenario than under the RCP8.5 scenario in several decades.

### Consideration of step-by-step adaptation pathways

Thus far we have developed adaptation pathways to maintain current yields as the timely case and prioritized cases, and we have discussed the effects of climate-change uncertainties, adaptation strategies, and emissions scenarios on the adaptation pathways. Here we look at them from the viewpoint of performing a series of adaptation processes. In the cases examined above, we assumed that a large amount of adaptation could be promptly implemented within a decade. However, we must consider the feasible rate of adaptation, because planned adaptation requires both significant investment and substantial time to be developed. We should also consider the lead time to implement an adaptation, which means the time lag between the planning and the implementation of adaptation. Here we consider step-by-step adaptation pathways, which are those described by gradual implementation of the adaptation and considering a lead time to implement an adaptation. In this case, full adaptation would not be achieved immediately under an abrupt climate change, such that adaptation forecasts will become important. In the following, we discuss the advantage of forecasting necessary adaptations.

**Figure 5:**
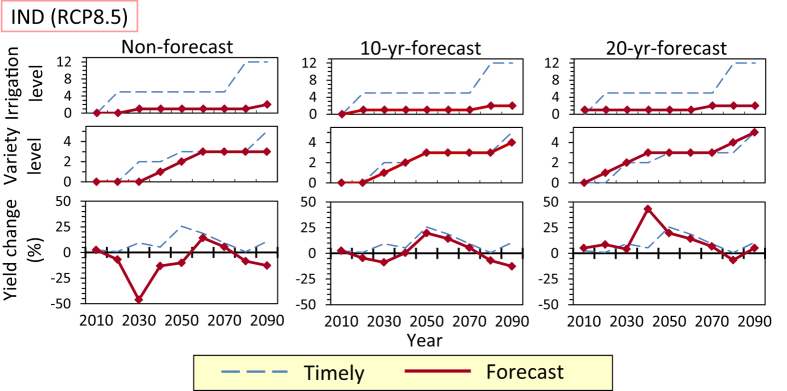
Adaptation pathways and yield change from the current level in the non-forecast case, 10-yr-forecast case, and 20-yr-forecast case in India (IND) under the RCP8.5 scenario of the MIROC-ESM projection. The pathways and yield change in the timely case are also shown.

If no forecast is conducted (i.e., adaptation to be implemented in the next decade is determined by the yield change in the current decade) and the amount of adaptation implemented is limited to one level per decade each for irrigation and crop variety, the adaptation pathways without prioritization result in the red dashed lines in Figs 1 and 2 (referred to as “Non-forecast” in the figures), under the MIROC-ESM projection. In India, Germany, and Pakistan, in which a large amount of adaptation (multiple increments of levels) is required over a short period in the timely case, a substantial yield decrease occurs in the non-forecast case; the negative impact of not forecasting is remarkable. However, the decreases in yield could be moderated if forecasts of 10 yr ahead were conducted (see Methods), even if the amount of adaptation implemented is limited (Fig. 5; 10-yr-forecast case). If a forecast to 20 yr ahead is available, further yield decreases may be moderated. In India, forecasting to 20 yr ahead could achieve a yield comparable to that in the timely case without implementing a high level of adaptation in a short period (Fig. 5; 20-yr-forecast case).

**Figure 6:**
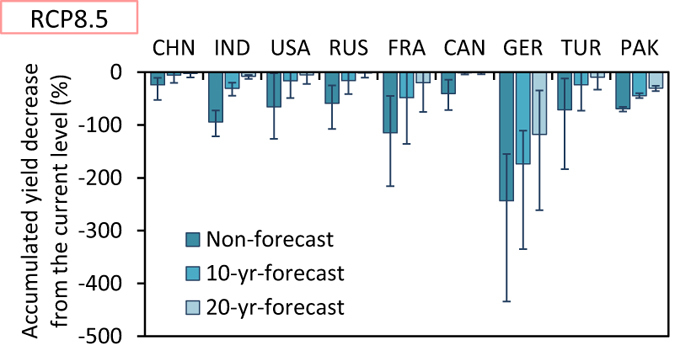
Accumulated yield decrease from the current level to the periods from the 2010 s to 2090 s in the forecast cases (non-forecast, 10-yr-forecast, and 20-yr-forecast cases) in the nine countries under the RCP8.5 scenario. The values are shown as relative to those in the timely case. The bar charts and the error bars are the averages and ranges of all the results based on different climate projections, respectively.

The efficacy of forecast is obvious in all nine countries and in all models and ensemble runs (Fig. 6). In China and Canada, the 10-yr-forecast case is nearly equal to the timely case in the multi-projection average of accumulated yield decrease during the 21st century (the 2010 s to 2090 s); the yield decrease from the current level could be avoided by forecasting 10 yr ahead. Even in India and Pakistan, the yield decrease could be moderated by forecasts. Therefore, forecasting the adaptation necessary in the future is important to achieve the benefit of the adaptation; in other words, the negative impacts of climate change could be moderated by implementing adaptations steadily according to forecasts of the necessary future adaptations, as compared to missing the appropriate timing to implement adaptations.

### Toward future research

In this study, we developed adaptation pathways in the current major wheat-producing countries, based on sequential introduction of the minimum adaptation measures necessary to maintain current wheat yields through the 21st century. The adaptation pathways revealed that the timing and intensity of adaptations required differs markedly among countries. The adaptation pathways are sensitive to both climate model uncertainty and natural variability of the climate system, and the degree of sensitivity differs among countries. In addition, we showed that forecasts of necessary adaptations are important to achieve the benefit of the adaptation pathways. Here we discuss several issues that must be considered to improve the approach proposed and adopted in this study, as well as the robustness of the results.

First, analyses synthesizing multiple climate projections will be required. Although we employed multiple climate models and ensemble runs to show the impacts of their uncertainties on the adaptation pathways, we need both more projections and more comprehensive analyses to identify robust adaptation pathways that can overcome the uncertainties.

Second, the availability and accessibility of adaptation levels assumed in this study include uncertainties. For example, the pathways in this study indicate when new crop varieties are required in addition to the existing crop varieties; however, it is uncertain whether the assumed new varieties will become a reality. Regarding irrigation, in the adaptation levels we included a large expansion of irrigation that may not be adopted due to economic limitations. The assumptions underlying the selectable adaptation levels need to be refined based on further empirical evidence.

Third, under the given adaptation sets we assumed that farmers are always able to attain the highest yield by optimizing planting dates and wheat varieties. However, the rate at which farmers will adapt is uncertain^20^. Better representation of farmers’ decision-making will be needed for more in-depth analyses. With respect to the effect of climate change on crop yield, we did not consider the impacts of pests and diseases (we considered only their indirect impacts by assuming that the risk of pests and diseases varies with the length of the growing period). Because pests and diseases affect crop yield and need to be adapted to, more precise analyses will be needed regarding this point.

Fourth, we also assumed that maintaining the current yield will be the target in the future as well, but the amount of yield required would vary with changes in socio-economic conditions (e.g., global food production necessary in 2050 is projected to be 60% higher than that of 2005/2007^21^). If an increase in the demand for food is expected due to population increase, for example, the adaptation pathways in this study may be insufficient. In addition, we accounted for neither adaptation costs nor technological progress in wheat production. Linkage with socio-economic projections will enable us to analyze adaptation pathways more comprehensively.

Although there is certainly room for improvement, this study is a useful first step in quantifying the temporal dimension of adaptation for global food production. Further research will enable us to develop more robust adaptation pathways, which would provide useful information for adaptation planning in each country.

## Methods

### Model and definition of adaptation levels

We calculated wheat yield with the M-GAEZ crop model^22^. In this model, yield for each grid is determined not only by biophysical conditions including climate (1° × 1°) but also by management conditions including water management (rain-fed or irrigated) and input level, which is a collective indicator incorporating factors that affect yield (e.g., fertilization level, technological development level)^22^. The input climatic variables are monthly mean temperature (°C), precipitation (mm/day), solar radiation (W/m^2^), wind speed (m/s), and diurnal temperature range (°C). The spatial resolution of final output yield is 2.5′ × 2.5′. To develop adaptation pathways, we aggregated grid-based yield into country-based yield.

The M-GAEZ model calculates wheat biomass based on the climatic variables, and it calculates wheat yield by imposing several constraints (e.g., a moisture constraint) and the harvest index on the biomass. The model also calculates the growing period of wheat based on the variety-specific growth cycle and climatic conditions, and judges whether the crop is able to grow or not based on the temperature profile in the grid cell during the growing period. In the M-GAEZ model, crops are assumed to be free from water stress when irrigation is applied; the negative impacts of a moisture deficit (related to evapotranspiration) can be fully avoided. (See Fischer *et al.*^23^ for further information of the model calculation.) Therefore, expanding the irrigated area as an adaptation has the potential to moderate a yield decrease. In addition, because the growth characteristics and suitable temperature profile differ among crop varieties, increasing the number of selectable varieties as an adaptation will increase the likelihood of attaining a higher yield.

At irrigation Level 0 (corresponding to no adaptation), the irrigated area is maintained as it is at present. To set irrigation from Levels 1 to 10, we increased the irrigated area ratio by 10% per adaptation level for crop lands where the present irrigated ratio is above zero. For Levels 11 and 12, in addition to increasing the ratio by 100% for crop lands where present irrigated ratio is above zero, we increased the ratio by 20% (for Level 11) and 50% (for Level 12) for presently non-irrigated crop lands (Supplementary Table S1).

The M-GAEZ model originally had 16 varieties of wheat, which are classified into four major cultivars (winter wheat, spring wheat, wheat in subtropics, and wheat in tropics), each of which has four minor varieties that differ in growth period. At variety Level 0, only four minor varieties of the current optimal cultivar (defined as the major cultivar that most frequently produced the highest yield from 1991 to 2000) are selectable to obtain the highest yield. At variety Level 1, all the 16 original varieties are selectable. To assume new heat-tolerant varieties, we conducted a sensitivity analysis by relaxing the limitations of high temperatures for the 16 varieties. From Levels 2 to 5, a set of 16 new varieties become selectable when the level rises to the next one. We removed the limitations of high temperatures at Level 5 (Supplementary Table S1); wheat is assumed to be free from the limitations of high temperatures within the suitable climatic zones. For all the calculations, the planting date was variable to produce the highest yield under the given adaptation set. The effect of CO_2_ fertilization was taken into account by applying multipliers to the yield according to the mean atmospheric CO_2_ concentration in each decade, in a way similar to Masutomi *et al.*^22^. (Note that the extent of the CO_2_ fertilization effect has large uncertainties.)

### Analyzed cases

In the timely case, the adaptation set that has the minimum yield change from the current level is chosen in each decade from among the adaptation sets in which yield change is maintained at or above zero. In case there are only adaptation sets in which the yield change is below zero, the set that minimizes the yield decrease from the current level is chosen. The prioritized cases (prioritize irrigation case and prioritize variety case) are the same as the timely case except that the prioritized adaptation option continues to be chosen until the yield changes at any adaptation levels fall below zero, while the level of the other option is fixed. If the yield changes at any levels are below zero, then the other option rises by one level, and the process is repeated. In case there are only adaptation sets in which the yield change is below zero, the set that minimizes the yield decrease from the current level is chosen regardless of the options. In the forecast cases (non-forecast case, 10-yr-forecast case, and 20-yr-forecast case), we assumed that the lead time to introduce an adaptation after planning is 10 yr. In the non-forecast case, the adaptation set required in a decade is implemented in the next decade, because the adaptation starts to be developed in the current decade. In the 10-yr-forecast case, the adaptation set required in the next decade is forecast (and starts to be developed) and is implemented in the next decade. In the 20-yr-forecast case, the adaptation set required in the two decades ahead is forecast (and starts to be developed) and is implemented in the next decade. Even though implementation of adaptation is not necessary two decades ahead, the adaptation set is implemented if it is required in the next decade. The non-forecast case, 10-yr-forecast case, and 20-yr-forecast case are limited in the amount of adaptation implemented to one level per decade. The assumed cases mentioned above are summarized in Supplementary Table S2. We show the conceptual diagram for the forecast cases in Supplementary Fig. S7.

### Data

Throughout the analyses, crop land area was fixed at the current condition^24^. The current irrigated area was based on MIRCA2000^25^. The calculation period was each decade (mean climate for 10 yr) for the present (1991–2000) and from the 2010 s (2011–2020) to 2090 s (2091–2100). We used three climate models: MIROC-ESM, MPI-ESM-LR, and CSIRO-Mk3-6-0, and we also employed three ensemble runs each of MPI-ESM-LR and CSIRO-Mk3-6-0. For MIROC-ESM, we used the projection under the RCP8.5 and RCP2.6 scenarios. For the other models and ensemble runs, we used the RCP8.5 scenario. The climate scenarios were downscaled to 1.0° × 1.0° gridded data by linear interpolation and used after bias correction with data derived from the Climate Research Unit^26^,^27^. The overall framework of developing pathways is presented in Supplementary Fig. S1. We assessed adaptation pathways for the top nine wheat-producing countries between 1991 and 2010^11^.

## Additional Information

**How to cite this article**: Tanaka, A. *et al.* Adaptation pathways of global wheat production: Importance of strategic adaptation to climate change. *Sci. Rep.* **5**, 14312; doi: 10.1038/srep14312 (2015).

## Supplementary Material

Supplementary Information

## References

[b1] Lobell D. B. *et al.* Prioritizing climate change adaptation needs for food security in 2030. Science 319, 607–610 (2008). 18239122 10.1126/science.1152339

[b2] Burke M. & Lobell D. in Climate Change and Food Security: Adapting Agriculture to a Warmer World (eds Lobell D. & Burke M.) Ch. 8, 133–153 (Springer, Heidelberg, 2010).

[b3] Olmstead A. L. & Rhode P. W. Adapting North American wheat production to climatic challenges, 1839–2009. Proc. Natl. Acad. Sci. USA 108, 480–485 (2011). 21187376 10.1073/pnas.1008279108 PMC3021086

[b4] Challinor A. J. *et al.* A meta-analysis of crop yield under climate change and adaptation. Nature Clim. Change 4, 287–291 (2014).

[b5] Hertel T. W. & Lobell D. B. Agricultural adaptation to climate change in rich and poor countries: Current modeling practice and potential for empirical contributions. Energy Econ. 46, 562–575 (2014).

[b6] Porter J. R. *et al.* in *Climate Change 2014: Impacts, Adaptation, and Vulnerability. Part A: Global and Sectoral Aspects*. Contribution of Working Group II to the Fifth Assessment Report of the Intergovernmental Panel on Climate Change (eds Field C. B. *et al.*) Ch. 7, 485–533 (Cambridge University Press, Cambridge and New York, 2014).

[b7] Haasnoot M., Middelkoop H., Offermans A., van Beek E. & van Deursen W. P. A. Exploring pathways for sustainable water management in river deltas in a changing environment. Clim. Change 115, 795–819 (2012).

[b8] Haasnoot M., Kwakkel J. H., Walker W. E. & ter Maat J. Dynamic adaptive policy pathways: A method for crafting robust decisions for a deeply uncertain world. Glob. Environ. Change 23, 485–498 (2013).

[b9] Maru Y. T., Smith M. S., Sparrow A., Pinho P. F. & Dube O. P. A linked vulnerability and resilience framework for adaptation pathways in remote disadvantaged communities. Glob. Environ. Change 28, 337–350 (2014).

[b10] Siebentritt M., Halsey N. & Stafford-Smith M. in Regional Climate Change Adaptation Plan for the Eyre Peninsula Ch. 7, 29–53 (the Eyre Peninsula Integrated Climate Change Agreement Committee, Australia, 2014). Available at: http://www.naturalresources.sa.gov.au/eyrepeninsula/projects-and-partners/climate-change (Date of access: 04/03/2015).

[b11] Food and Agriculture Organization of the United Nations (FAO), FAOSTAT. http://faostat3.fao.org/faostat-gateway/go/to/home/E (2013). (Date of access: 23/10/2013).

[b12] Watanabe S. *et al.* MIROC-ESM 2010: Model description and basic results of CMIP5-20c3m experiments. Geosci. Model Dev. 4, 845–872 (2011).

[b13] Giorgetta M. A. *et al.* Climate and carbon cycle changes from 1850 to 2100 in MPI-ESM simulations for the Coupled Model Intercomparison Project phase 5. J. Adv. Model. Earth Syst. 5, 572–597 (2013).

[b14] Jeffrey S. *et al.* Australia’s CMIP5 submission using the CSIRO-Mk3.6 model. Aust. Meteor. Ocean. J. 63, 1–13 (2013).

[b15] Taylor K. E., Stouffer R. J. & Meehl G. A. An overview of CMIP5 and the experiment design. Bull. Amer. Meteor. Soc. 93, 485–498 (2012).

[b16] Hawkins E. & Sutton R. The potential to narrow uncertainty in regional climate predictions. Bull. Amer. Meteor. Soc. 90, 1095–1107 (2009).

[b17] Deser C., Knutti R., Solomon S. & Phillips A. S. Communication of the role of natural variability in future North American climate. Nature Clim. Change 2, 775–779 (2012).

[b18] Riahi K. *et al.* RCP 8.5—A scenario of comparatively high greenhouse gas emissions. Clim. Change 109, 33–57 (2011).

[b19] van Vuuren D. P. *et al.* RCP2.6: Exploring the possibility to keep global mean temperature increase below 2°C. Clim. Change 109, 95–116 (2011).

[b20] Moore F. C. & Lobell D. B. Adaptation potential of European agriculture in response to climate change. Nature Clim. Change 4, 610–614 (2014).

[b21] Alexandratos N. & Bruinsma J. in World Agriculture towards 2030/2050: the 2012 revision. ESA Working paper No. 12-03. Ch. 1, 1–22 (Food and Agriculture Organization of the United Nations (FAO), Rome, 2012).

[b22] Masutomi Y., Takahashi K., Harasawa H. & Matsuoka Y. Impact assessment of climate change on rice production in Asia in comprehensive consideration of process/parameter uncertainty in general circulation models. Agric. Ecosyst. Environ. 131, 281–291 (2009).

[b23] Fischer G., van Velthuizen H., Shah M. & Nachtergaele F. in Global Agro-ecological Assessment for Agriculture in the 21st Century: Methodology and Results. RR-02-02. Ch. 2–4, 6–64 (International Institute for Applied Systems Analysis and Food and Agriculture Organization of the United Nations, Laxenburg, 2002).

[b24] Monfreda C., Ramankutty N. & Foley J. A. Farming the planet: 2. Geographic distribution of crop areas, yields, physiological types, and net primary production in the year 2000. Glob. Biogeochem. Cycles 22, GB1022 (2008).

[b25] Portmann F. T., Siebert S. & Döll P. MIRCA2000—Global monthly irrigated and rainfed crop areas around the year 2000: A new high-resolution data set for agricultural and hydrological modeling. Glob. Biogeochem. Cycles 24, GB1011 (2010).

[b26] New M., Hulme M. & Jones P. Representing twentieth-century space–time climate variability. Part 1. Development of a 1961–90 mean monthly terrestrial climatology. J. Clim. 12, 829–856 (1999).

[b27] Mitchell T. D. & Jones P. D. An improved method of constructing a database of monthly climate observations and associated high-resolution grids. Int. J. Climatol. 25, 693–712 (2005).

