# Supplementary material for: Adaptation pathways of global wheat production: Importance of strategic adaptation to climate change
Source: Sci Rep. 2015 Sep 16;5:14312. doi: 10.1038/srep14312 (PMC4571615; doi:10.1038/srep14312)
Supplement: Supplementary Information [file srep14312-s1.pdf]

Supplementary Information

Adaptation pathways of global wheat production: Importance of strategic adaptation to climate change

Akemi Tanaka[1], Kiyoshi Takahashi[1], Yuji Masutomi[2], Naota Hanasaki[3], Yasuaki Hijioka[1], Hideo Shiogama[3], and Yasuhiro Yamanaka[4]

[1]Center for Social and Environmental Systems Research, National Institute for Environmental Studies, 16-2 Onogawa, Tsukuba, Ibaraki 305-8506, Japan

[2]College of Agriculture, Ibaraki University, 3-21-1 Chuo, Ami, Inashiki, Ibaraki 300-0393, Japan

[3]Center for Global Environmental Research, National Institute for Environmental Studies, 16-2 Onogawa, Tsukuba, Ibaraki 305-8506, Japan

[4]Faculty of Environmental Earth Science, Hokkaido University, N10 W5, Kita-ku, Sapporo, Hokkaido 060-0810, Japan

---

Supplementary Tables

Supplementary Table S1 | Adaptation levels

Level	Description
Irrigation levels*	
0	No adaptation (fixed to current irrigated area ratio)
1 to 10	Increasing irrigated area ratio by X% for crop lands where irrigated ratio in the present > 0 (X=10 for Level 1, 20 for Level 2, …, 100 for Level 10)
11	Increasing irrigated area ratio by 100% for crop lands where irrigated ratio in the present > 0, and increasing by 20% for presently non-irrigated crop lands
12	The same as Level 11 except increasing by 50% for presently non-irrigated crop lands
Crop variety levels	
0	No adaptation (only 4 minor varieties of the present optimal cultivar† [defined in the M-GAEZ model] are selectable)
1	Original 16 varieties defined in the M-GAEZ model are selectable
2 to 4	A set of 16 new heat-tolerant varieties (assumed by gradually relaxing temperature limitations of high temperatures in the M-GAEZ model) added to the selectable varieties in the previous level are selectable
5	A set of 16 new heat-tolerant varieties (assumed by removing temperature limitations of high temperatures in the M-GAEZ model) added to the selectable varieties in Level 4 are selectable

*Upper limit of irrigated area ratio is 100%.

†The optimal cultivar is set separately for rain-fed crop lands and irrigated crop lands.

Supplementary Table S2 | Assumptions of implementing adaptation sets to develop adaptation pathways

Case	Description	Limitation of implementing adaptation	Prioritized adaptation option	Forecast
Timely	• An adaptation set that has the minimum yield change is chosen from among the sets in which yield change is at or above zero<br>• If there are only sets in which yield change is below zero, a set that minimizes the yield decrease is chosen<br>• The adaptation set required in the current decade is implemented immediately in the current decade	none	none	-
Prioritized cases				
Prioritize irrigation	The same as the timely case, except that the adaptation of irrigation is preferentially chosen	none	irrigation	-
Prioritize variety	The same as the timely case, except that the adaptation of crop variety is preferentially chosen	none	crop variety	-
Forecast cases				
Non-forecast	The same as the timely case, except that:<br>• The adaptation set required in the current decade is implemented in the next decade<br>• Adaptation levels actually implemented are limited to one level per decade	one level per decade	none	none
10-yr-forecast	The same as the timely case, except that:<br>• The adaptation set to be selected in the next decade is forecast and implemeted in the next decade<br>• Adaptation levels actually implemented are limited to one level per decade	one level per decade	none	10 yr ahead
20-yr-forecast	The same as the timely case, except that:<br>• The adaptation set to be selected in the two decades ahead is forecast and implemeted in the next decade<br>• Adaptation levels actually implemented are limited to one level per decade	one level per decade	none	20 yr ahead

Supplementary Figures

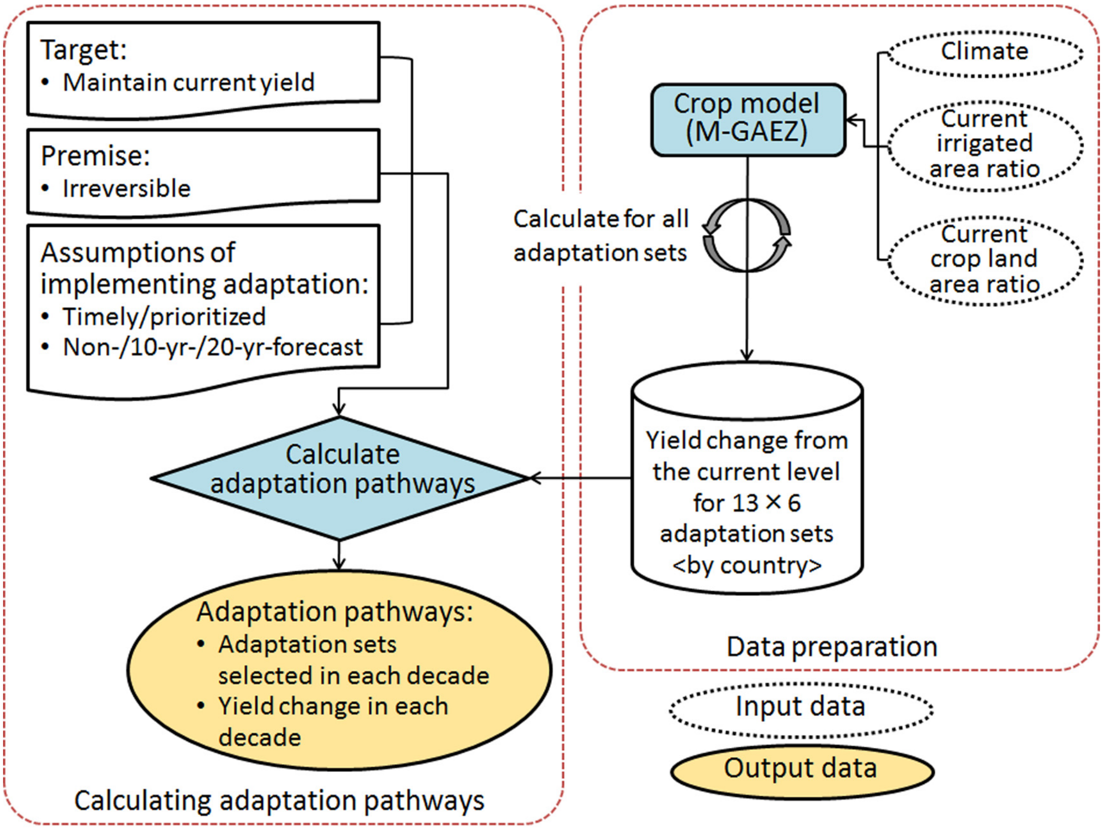

Supplementary Figure S1 | Framework of developing adaptation pathways.

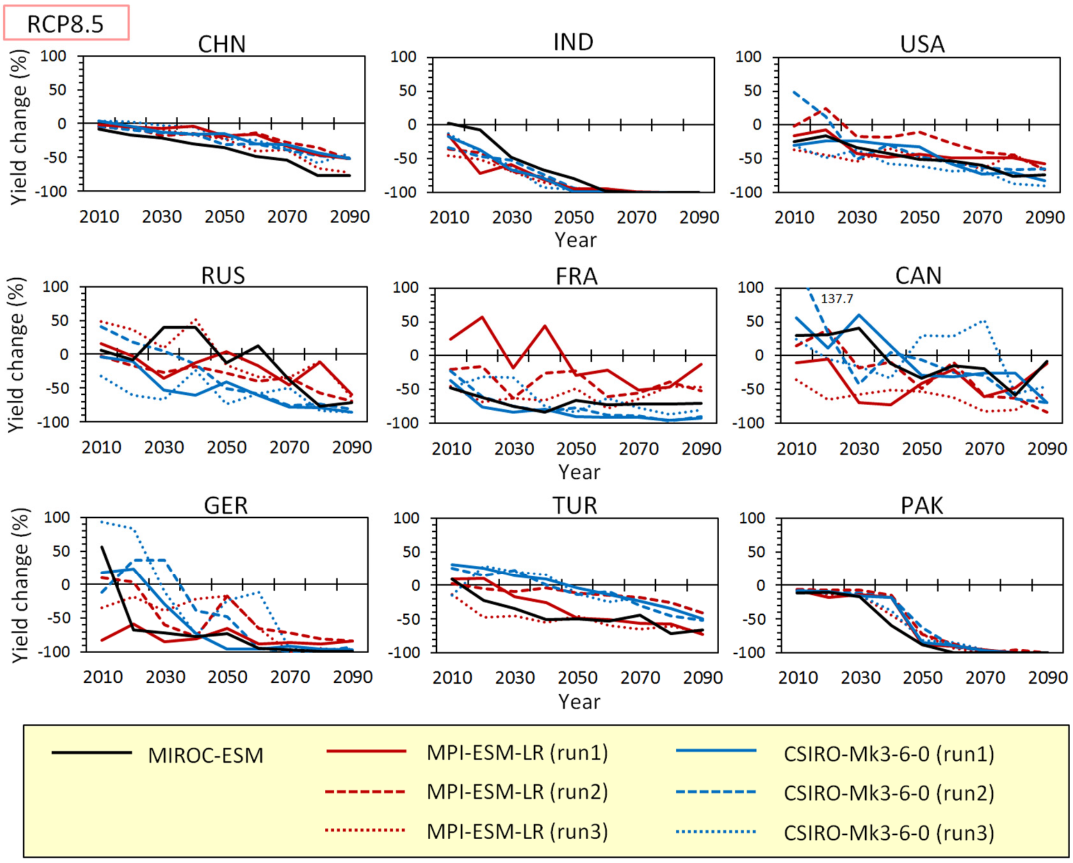

Supplementary Figure S2 | Wheat yield change from the current level without adaptation (i.e., adaptation Level 0 for both irrigation and crop variety) in China (CHN), India (IND), the United States (USA), Russia (RUS), France (FRA), Canada (CAN), Germany (GER), Turkey (TUR), and Pakistan (PAK), under the RCP8.5 scenario. The descriptions "run1", "run2", and "run3" correspond to r1i1p1, r2i1p1, and r3i1p1 of the CMIP5 simulations, respectively.

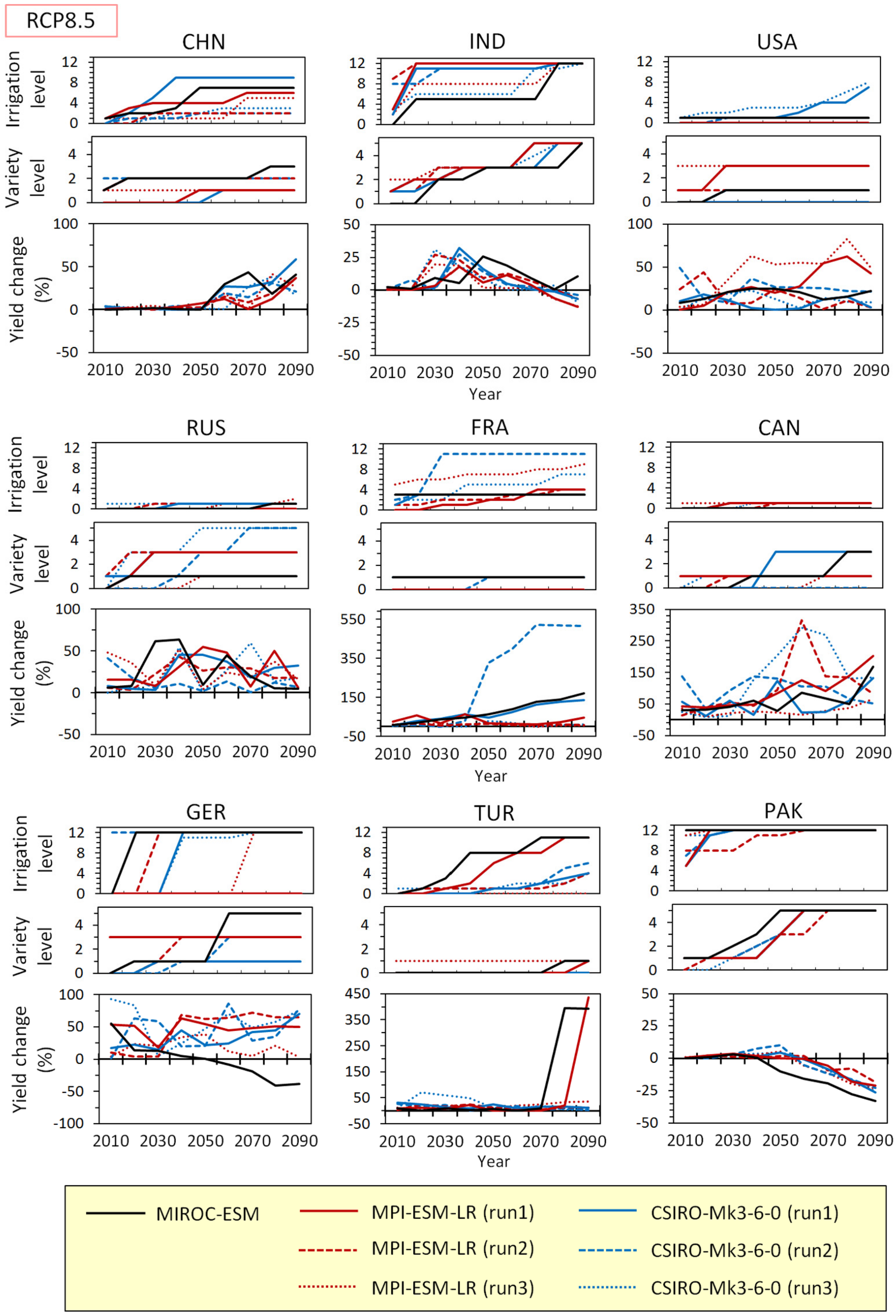

Supplementary Figure S3 | Adaptation pathways and yield change from the current level in the timely case of all the climate projections in China (CHN), India (IND), the United States (USA), Russia (RUS), France (FRA), Canada (CAN), Germany (GER), Turkey (TUR), and Pakistan (PAK), under the RCP8.5 scenario. The descriptions “run1”, “run2”, and “run3” correspond to r1i1p1, r2i1p1, and r3i1p1 of the CMIP5 simulations, respectively.

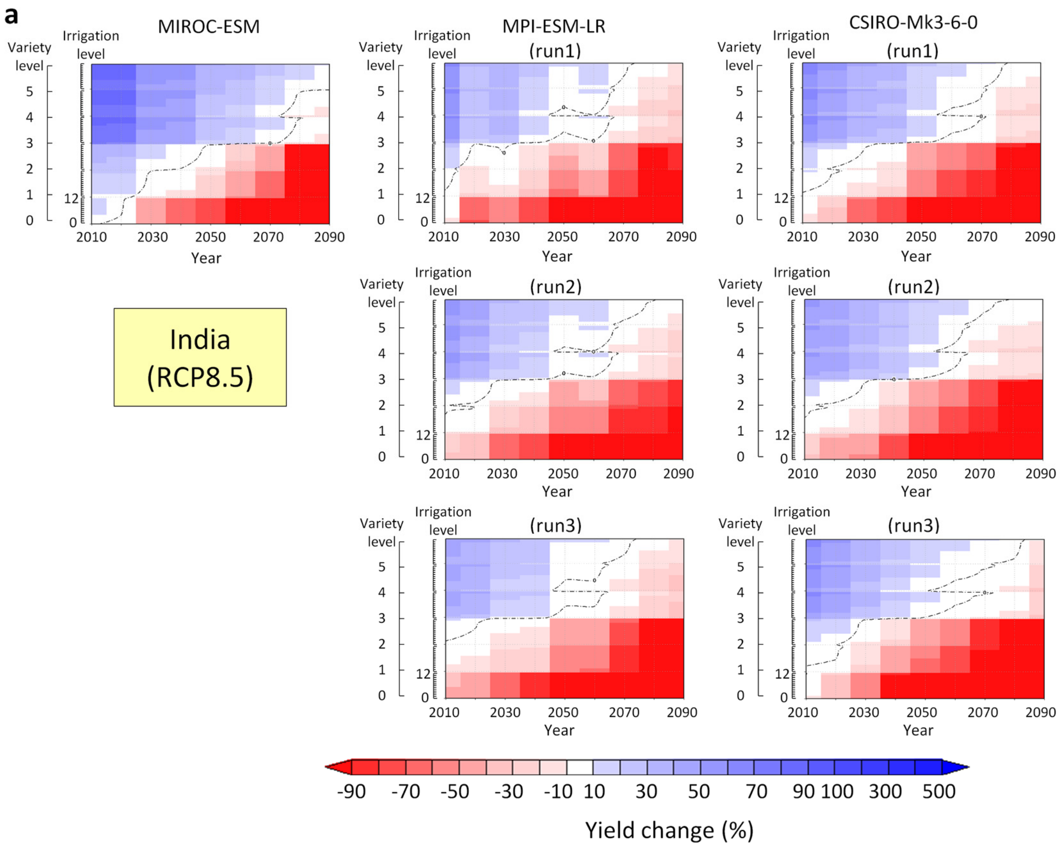

Supplementary Figure S4 | Time evolution of yield change from the current level for all adaptation sets (13 irrigation × 6 variety) under the RCP8.5 scenario of all the climate projections. The thin black broken lines represent 0% change. The descriptions "run1", "run2", and "run3" correspond to r1i1p1, r2i1p1, and r3i1p1 of the CMIP5 simulations, respectively. **a**, India. **b**, Germany.

(Supplementary Figure S4; continued)

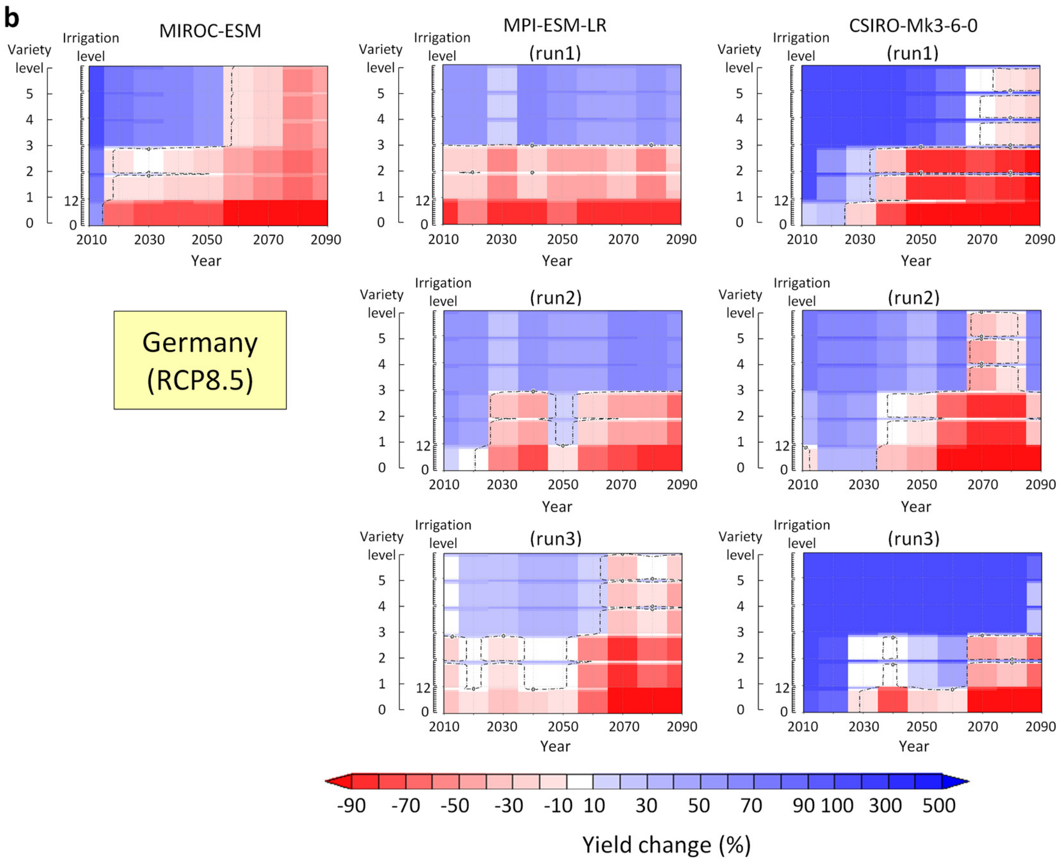

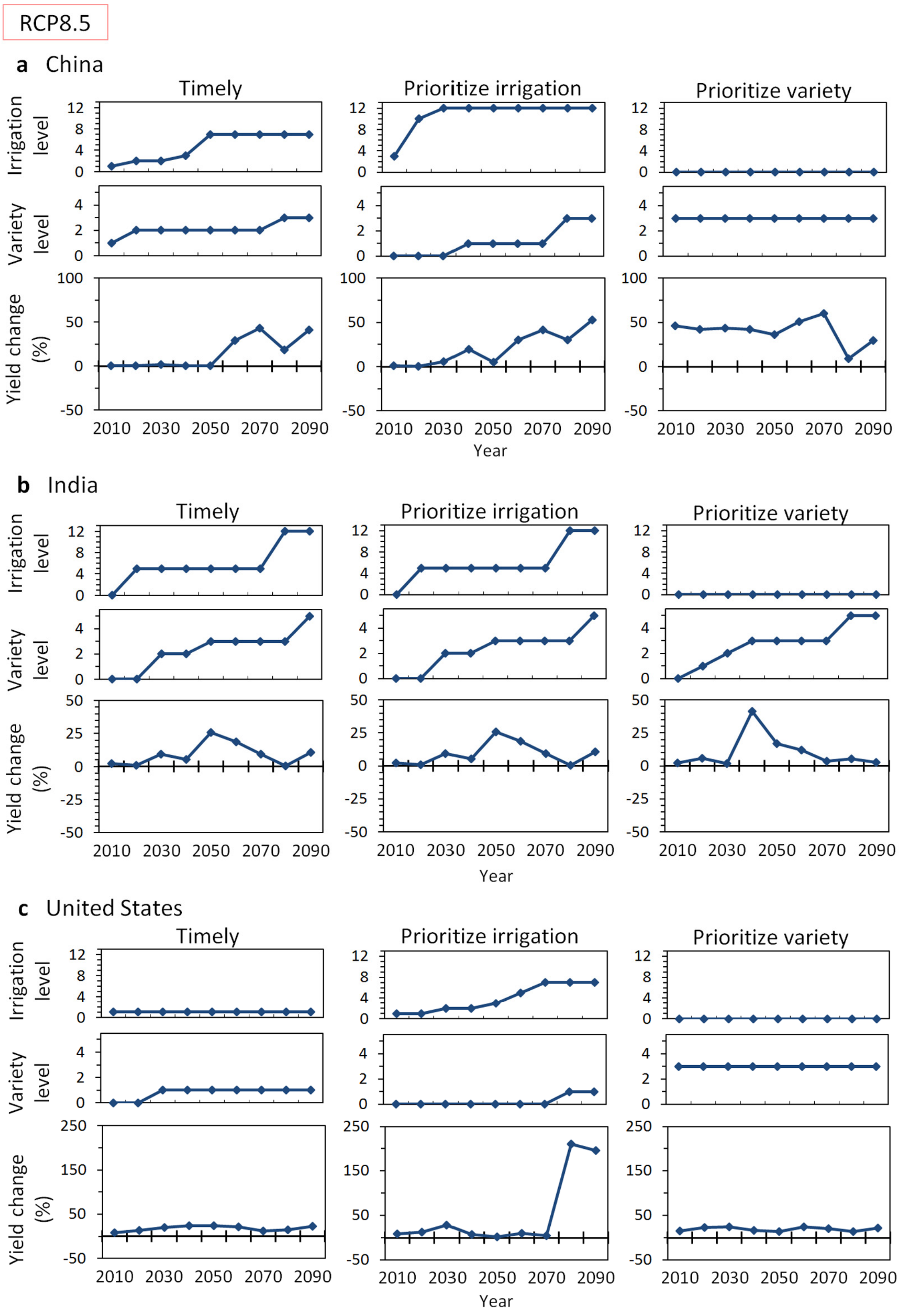

Supplementary Figure S5 | Adaptation pathways and yield change from the current level in the timely case, prioritize irrigation case, and prioritize variety case in the nine countries under the RCP8.5 scenario of the MIROC-ESM projection.

(Supplementary Figure S5; continued)

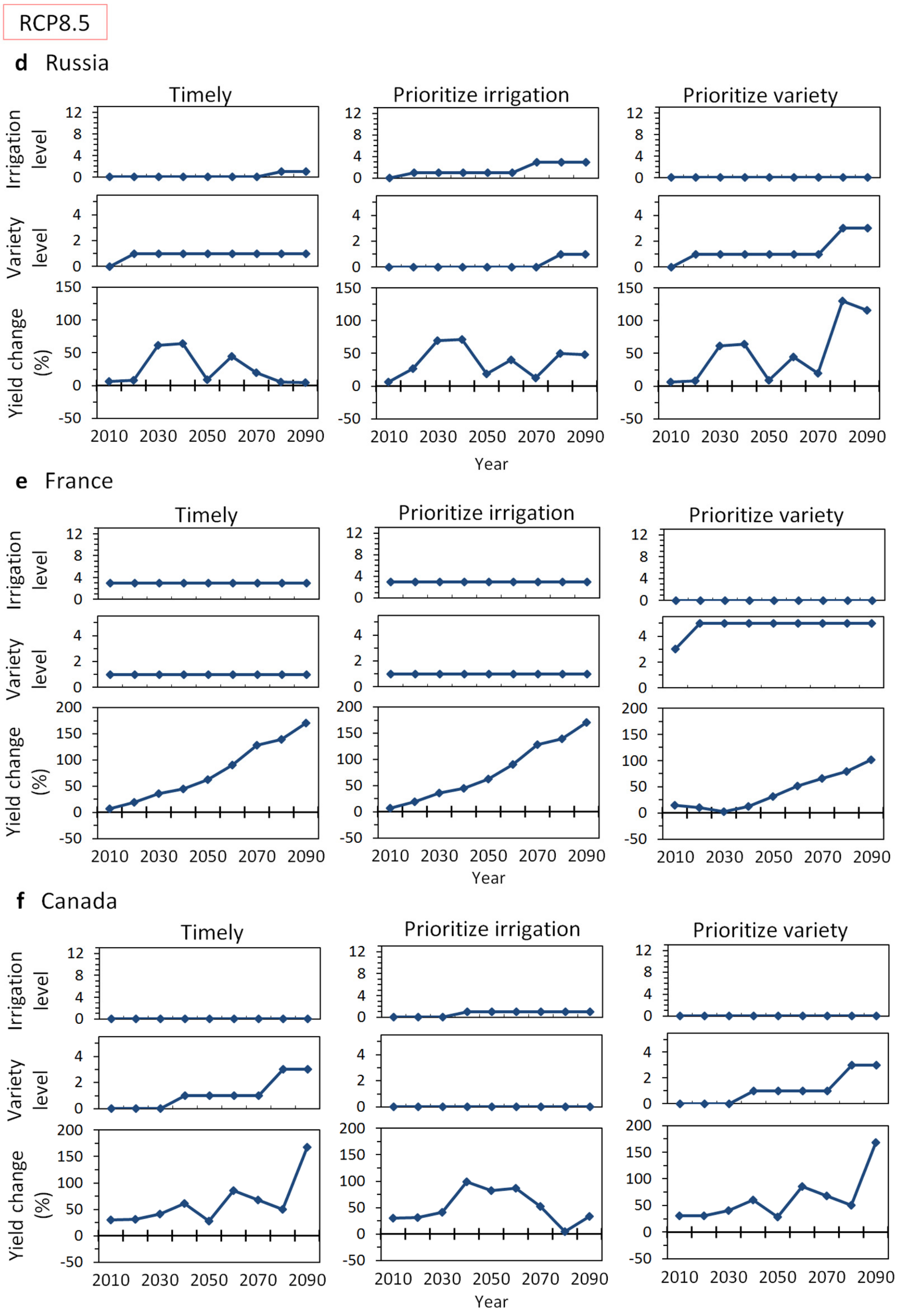

(Supplementary Figure S5; continued)

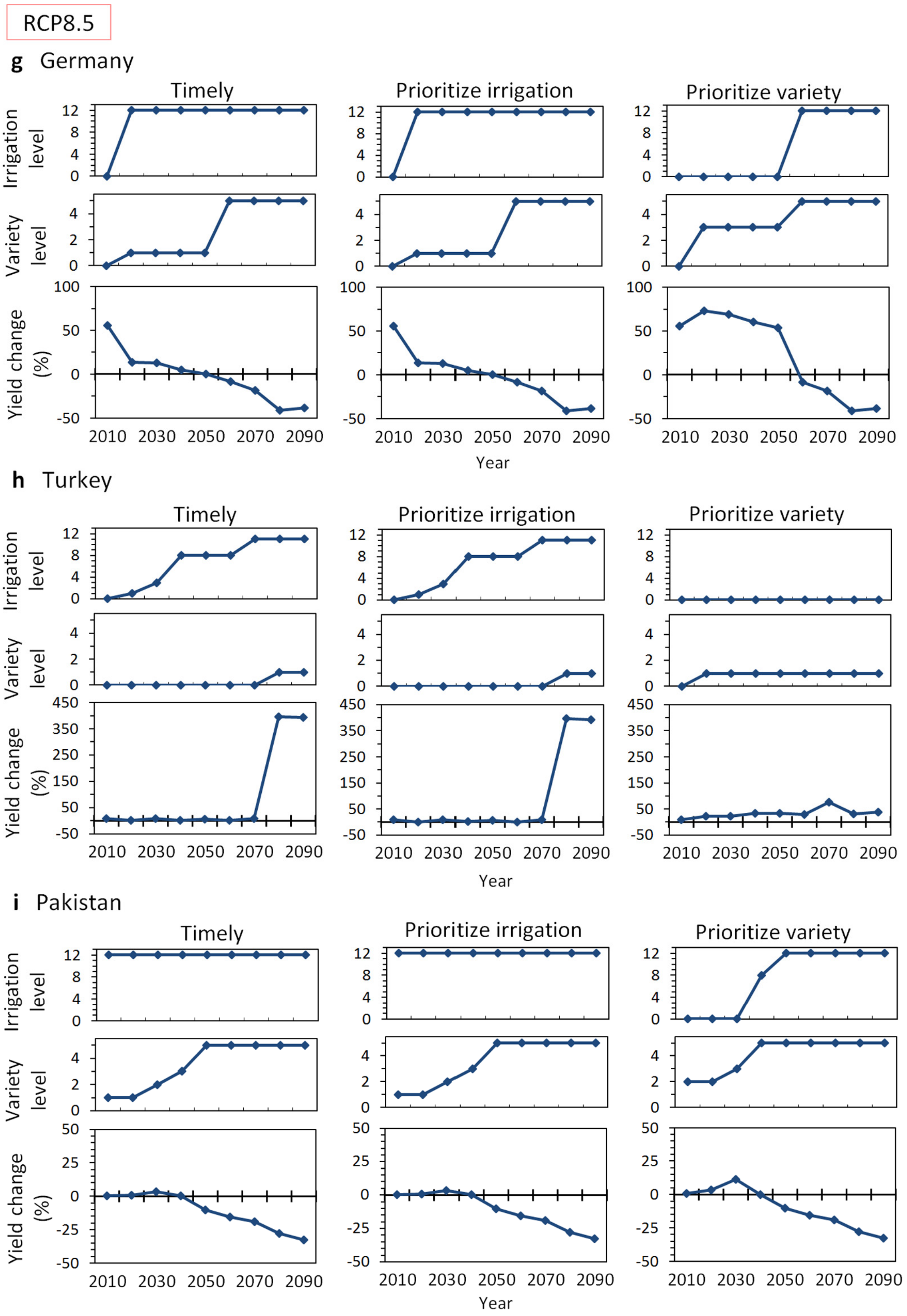

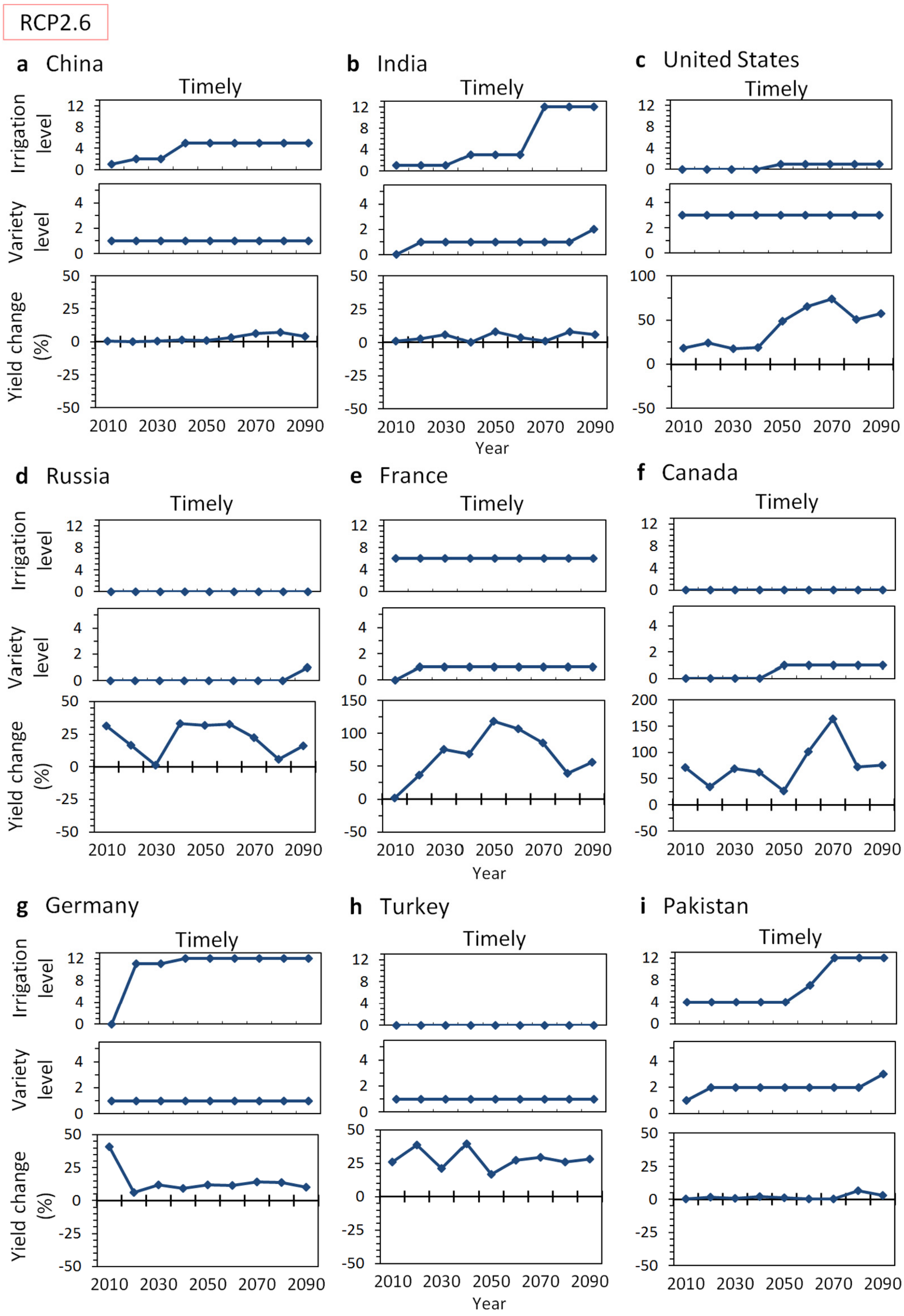

Supplementary Figure S6 | Adaptation pathways and yield change from the current level in the timely case in the nine countries under the RCP2.6 scenario of the MIROC-ESM projection.

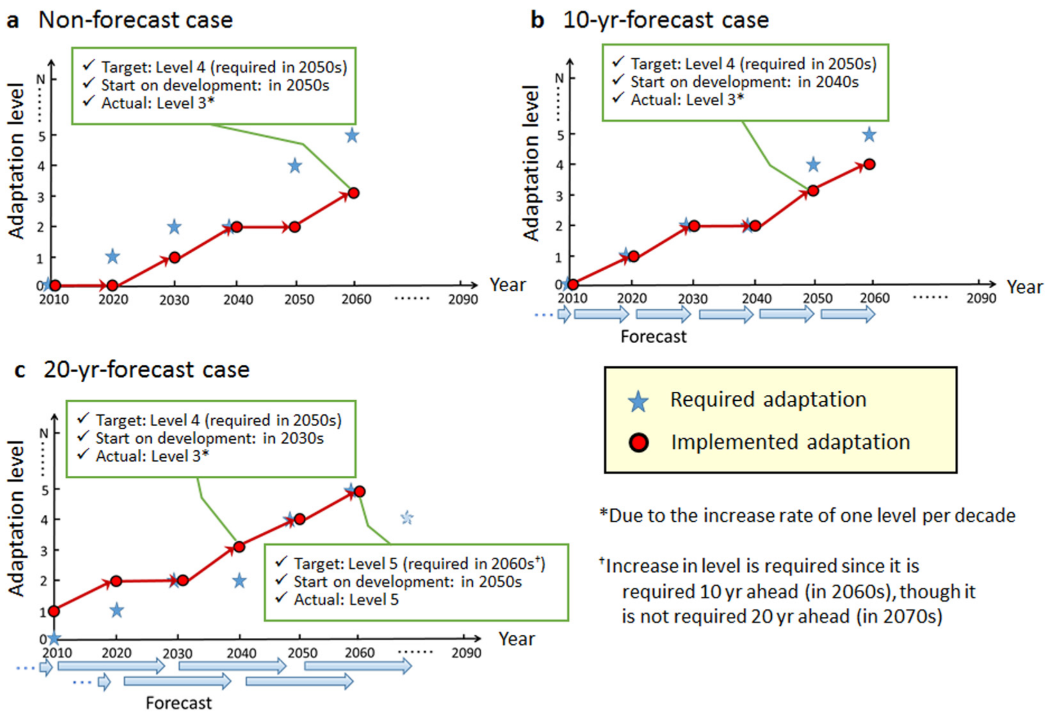

Supplementary Figure S7 | Conceptual diagram of the forecast cases (non-forecast case, 10-yr-forecast case, and 20-yr-forecast case).
